# Psychometric validation of the Italian Rehabilitation Complexity Scale-Extended version 13

**DOI:** 10.1371/journal.pone.0178453

**Published:** 2017-10-18

**Authors:** Francesca Roda, Maurizio Agosti, Andrea Merlo, Maurizio Maini, Francesco Lombardi, Claudio Tedeschi, Maria Grazia Benedetti, Nino Basaglia, Mara Contini, Domenico Nicolotti, Rodolfo Brianti, on behalf of GRECo

**Affiliations:** 1 Department of Medicine and Surgery, Unit of Neuroscience, University of Parma, Parma, Italy; 2 Rehabilitation Medicine Service, Rehabilitation Geriatrics Department of the NHS-University Hospital of Parma, Parma, Italy; 3 Motion Analysis Laboratory, Department of Rehabilitation, "S. Sebastiano" Hospital of Correggio, NHS Local Agency of Reggio Emilia, Reggio Emilia, Italy; 4 “San Giacomo” Hospital, Ponte dell’Olio, Piacenza, Italy; 5 Neurorehabilitation Service, "S. Sebastiano" Hospital of Correggio, NHS Local Agency of Reggio Emilia, Reggio Emilia, Italy; 6 Physical medicine and Rehabilitation Unit – Neuromotor Department, IRCCS "Arcispedale Santa Maria Nuova" of Reggio Emilia, Reggio Emilia, Italy; 7 Physical Medicine and Rehabilitation Unit, “Rizzoli” Orthopaedics Hospital, Bologna, Italy; 8 Department of Neuroscience and Rehabilitation, University Hospital of Ferrara, Ferrara, Italy; 9 Extensive Orthopaedic Rehabilitation Unit, Department of Medicine, Borgo Val Di Taro Hospital, NHS Local Agency of Parma, Parma, Italy; 10 Intensive Rehabilitation Medicine Spinal Unit, Emergency Medicine Department, Villanova d’Arda Hospital, NHS Local Agency of Piacenza, Piacenza, Italy; IRCCS E. Medea, ITALY

## Abstract

In Italy, at present, a well-known problem is inhomogeneous provision of rehabilitative services, as stressed by MoH, requiring appropriate criteria and parameters to plan rehabilitation actions. According to the Italian National Rehabilitation Plan, Comorbidity, Disability and Clinical Complexity should be assessed to define the patient’s real needs. However, to date, clinical complexity is still difficult to measure with shared and validated tools. The study aims to psychometrically validate the Italian Rehabilitation Complexity Scale-Extended v13 (RCS-E v13), in order to meet the guidelines requirements. An observational multicentre prospective cohort study, involving 8 intensive rehabilitation facilities of the Emilia-Romagna Region and 1712 in-patients, [823 male (48%) and 889 female (52%), mean age 68.34 years (95% CI 67.69–69.00 years)] showing neurological, orthopaedic and cardiological problems, was carried out. The construct and concurrent validity of the RCS-E v13 was confirmed through its correlation to Barthel Index (disability) and Cumulative Illness Rating Scale (comorbidity) and appropriate admission criteria (not yet published), respectively. Furthermore, the factor analysis indicated two different components (“Basic Care or Risk—Equipment” and “Medical—Nursing Needs and Therapy Disciplines”) of the RCS-E v13.

In conclusion, the Italian RCS-E v13 appears to be a useful tool to assess clinical complexity in the Italian rehab scenario case-mix and its psychometric validation may have an important clinical rehabilitation impact allowing the assessment of the rehabilitation needs considering all three dimensions (disability, comorbidity and clinical complexity) as required by the Guidelines and the inhomogeneity could be reduced.

## Introduction

According to the Italian National Plan for Rehabilitation (INPR) of 2011 [1], the Italian National Health Service (NHS) provides, in post-acute in-patients settings, for 3 levels of different Physical & Rehabilitation Medicine (PRM) interventions: intensive, intensive at high specialisation units and long-term or extensive care. Each Level should be based on the patient’s specific needs. A small number of people have very complex needs and require a higher level of highly specialised PRM care (e.g. post-acute spinal cord injury patients). A larger number of people also require specialist rehabilitation in similar settings, but in less specialised units (Intensive Level) for conditions such as some musculoskeletal trauma and neurological problems (e.g. Stroke).

In Italy, at present, especially at the Intensive rehabilitation level, the provision of rehabilitative services is not homogeneous and this is a well-known problem, as illustrated in an analysis of information flows disseminated by the Italian Ministry of Health with reference to the years 2010–2012 [2]. The report stresses the need to use appropriate criteria and parameters in the planning of rehabilitation actions. The same contents are also emphasised in Article 3 of the recent “Patto per la Salute 2014–2016” [3], reflecting the current problem of the growing economic impact of rehabilitation care and the cost-effectiveness analysis.

The INPR^1^ clearly specifies that Comorbidity, Disability and Clinical Complexity should be assessed to define the patient’s real needs and to plan the appropriate rehabilitation setting.

Whereas instruments such as the Barthel Index (BI) [4,5] and the Cumulative Illness Rating Scale (CIRS) [6] are typically used to rate disability and comorbidity, clinical complexity remains difficult to define.

Some European Countries, the US, Canada and Australia consider physical dependency and comorbidity also as indicators of clinical complexity of patients [7]. The available literature on rehabilitation suggests the use of various instruments to effectively evaluate Complexity in the rehabilitation domain [8–15]. Nevertheless, the scale that appears to be the most reliable and easy to use in order to evaluate the complexity dimension is the English Rehabilitation Complexity Scale (RCS).

The scale has been designed to provide a simple measure of rehabilitation complexity needs and interventions across the rehabilitation services and to determine the cost of the rehabilitation programmes delivered [16]

The preliminary version of the RCS^16^ assessed basic care, specialist nursing needs, therapy (in terms of time) and medical intervention, in order to take into account a description of the patient’s situation from a multi-professional (both a medical and a non-medical) point of view. A few years later the scale was revised [17] as RCS version 2, in which the therapy domain has been divided into two subscales reflecting the number of disciplines and the overall intensity of treatment [17]. The total score range was 0–15. However, clinicians working with the scale reported problems in identifying some aspects characterising patients with very complex needs, such as needs for special equipment. An Extended version of the RCS was developed to address these deficiencies [18]. The RCS-E version 12 [18] included 5 domains, Basic Care (0–4), Nursing Needs (0–3), Therapy (TD: 0–4, TI (0–4), Medical intervention (0–3) and Equipment (0–2) with a total score range from 0 to 20.

The latest version of the scale—is RCS-E version 13 –is currently used in the UK Rehabilitation Outcome Collaborative (UKROC) data set [19], a case-mix measure to strike a cost-effective balance between outcome and service cost, particularly for highly complex cases.

The RCS-E v13 encompasses the original 4 domains (C-N-M-T), which have been supplemented with a higher number of explanatory items of medical (0–4) and nursing (0–4) domains to assess higher levels of complex interventions. Furthermore, a new sub-domain “Risk” (0–4) was introduced to take account also of any need for supervision related to patients’ cognitive and behavioural aspects. Along with the Cure domain, a new domain called “Care or Risk” (0–4) was created. Where risk is measured, for the assessment both CARE and RISK shall be measured and the highest score shall be used.

Therefore, the English final version of the tool provides for 5 domains and has a score ranging from 0 to 22; it has an added value over the previous version in detecting patients with highly complex rehabilitation needs.

The RCS instrument proved practical and not time-consuming (administration time 5–10 min max). Furthermore, thanks to its psychometric properties [17–18], it seems to be an excellent tool in supporting the decision to refer patients to rehabilitation. Recent studies reached the same positive conclusion validating the Danish RCS-E version 13 [20] and the Italian RCS-E version 12 [21].

The cross-cultural adaptation of the Italian RCS-E v13 also demonstrated that it is a sensitive and reliable tool that seems suitable for measuring clinical complexity in Italian intensive rehabilitation units, as reported in Rodà et al 2015 [22]. This preliminary study was designed in strict compliance with the relevant international guidelines, face validity and test-retest reliability were carried out to evaluate the comprehensibility and goodness of fit of the updated scale; it was conducted in three different rehabilitation units of the Emilia Romagna region (Northern Italy), involving 10 expert physicians and recruited 51 Intensive rehabilitation in-patients.

The aim of this study is to provide the first Italian RCS-E v13 validation by correlating the score of the scale with BI and CIRS, plus a list of appropriate admission criteria (see methodology) in three different categories of rehabilitation intensive in-patients [Neurological Patients (NP), Cardiological Patients (CP), Orthopaedic Patients (OP)] in order to support the use of the tool in Italy and to meet the INPR requirements.

## Materials and methods

### Study design, setting, sample and procedure

This is an observational multicentre prospective cohort study that did not change the everyday routine clinical practice but required collection of sensitive data for research purposes in compliance with the local Guidelines [23] and with the Declaration of Helsinki.

The study protocol was approved by the Bioethics Committee (CE) of the University Hospital of Parma (ref. CEPR-ProtN1175) and by the ethics committees (the ethics committee of the Reggio Emilia Province about "S. Sebastiano" Hospital of Correggio and IRCCS "Arcispedale Santa Maria Nuova" of Reggio Emilia; the internal ethics committee of the “San Giacomo” Hospital, Ponte dell’Olio, the internal ethics committee of the “Rizzoli” Orthopaedics Hospital, Bologna and finally the ethics committee of the NHS Local Agency of Piacenza for the Villanova d’Arda Hospital) of the centres taking part in the study. All the participants were informed of the implications of the study and signed an informed consent document before enrolling. All the relevant study documents were kept in the Inspector Site File and in the Trial Master File.

8 intensive rehabilitation facilities (Table 1) of various types of agencies, both National Health System (NHS) and private ones, as well as Research Hospitals, covering a large part of the Emilia Romagna Region (RER), participated in the research.

**Table 1:** Rehabilitation facilities participating in the research.

1.	Rehabilitation Medicine Service, Rehabilitation Geriatrics Department of the NHS-University Hospital of Parma
2.	“San Giacomo” Hospital, Ponte dell’Olio, Piacenza
3.	Neurorehabilitation Service, "S. Sebastiano" Hospital of Correggio, NHS Local Agency of Reggio Emilia
4.	Physical medicine and Rehabilitation Unit—Neuromotor Department, IRCCS "Arcispedale Santa Maria Nuova" of Reggio Emilia, Reggio Emilia
5.	Physical Medicine and Rehabilitation Unit, “Rizzoli” Orthopaedics Hospital, Bologna
6.	Department of Neuroscience and Rehabilitation, University Hospital of Ferrara
7.	Extensive Orthopaedic Rehabilitation Unit, Department of Medicine, Borgo Val Di Taro Hospital, NHS Local Agency of Parma
8.	Intensive Rehabilitation Medicine Spinal Unit, Emergency Medicine Department, Villanova d’Arda Hospital, NHS Local Agency of Piacenza

All the adult neurological, cardiologic and orthopaedic patients admitted to the intensive rehabilitation facilities from June 2014 to August 2015 were enrolled, for a total of 1712.

Before starting to collect the study data, at least one Rehabilitation Medicine Specialist (RMS) of the 8 participating rehabilitative facilities was trained in the administration of the experimental scales (RCS-E v13, CIRS, BI) and in the experimental procedures to ensure research efficiency. Statistical correlation analysis was then performed to assess training effectiveness.

Moreover, an electronic Case Report Form (eCRF)—GCP-compliant and compatible with the Italian NHS Information systems—was developed to collect the relevant experimental information.

Within the 72 hours after admission, i.e. the time required to evaluate the patients’ needs and plan the rehabilitation intervention, the trained RMSs administered the three scales (spending about 20 min for each patient: RCS-E v13: 5 min, CIRS 10 min, BI 5 min) and recorded the scores, together with the usual clinical information, in the medical records.

Three trained MD Detectors (MDDs), recruited specifically for this project and not involved in patient admission, were provided with laptops and authorised to move around the centres to enter the data from the medical records to the eCRF.

### Variables/Measures

The variables used in the study were the Rehabilitation Complexity Scale Version 13 (RCS-E v13), the Barthel Index (BI), the Cumulative Illness Rating Scale (CIRS) and the Appropriate Admission Criteria (data not yet published).

The RCS-E v13 is a 22-point measure with 5 domains (see Rodà et al 2015): Basic Care or Risk (C or R: 0–4); Nursing (N: 0–4); Medical Needs (M: 0–4); Therapy Needs (Including the Number of Disciplines: TD and the Intensity: TI. TD+TI: 0–8); Equipment Needs (E:0–2). The higher the score, the more complex the needs are.

The BI is a 10-item ordinal scale that measures self-care, sphincter management, transfers and locomotion. Scores range from 0 to 100, in 5 steps, and the higher the score, the more independent the patient is.

The CIRS resulted in 14 categories, assessing the pathology and impairment of major organ systems and also psychological, metabolic, neurological and musculoskeletal aspects with a 0 (No problem) to 4 (Extremely severe problem) grading scale in each category. Several indices were derived from the CIRS: the total score (CTs) may theoretically vary from 0 to 56, although a high score is not compatible with life; the severity index (SI) is the mean of the scores of the first 13 categories, excluding the psychiatric one; and finally the comorbidity index (CI) as the number of categories with a score of 2 or greater, including the psychiatric one.

The list of Appropriate Admission Criteria in intensive rehabilitation facilities encompasses 14 items addressing functional ability/disability (e.g. mobility, self-managing, etc.), nursing care, medical and rehabilitation treatments needs, social and prognostic aspects (See S1 Appendix).

The list is the result of 6-month long stepwise work carried out through a consensus Delphi Panel Method [24]. The first step was collecting the admission criteria of the 8 centres involved in this study to prepare and provide the expert panellists with consecutive questionnaires, using a computerised process.

The first questionnaire consisted of 13 questions, some of which were broken down into sub-items, for a total of 29 items of a general nature. Patients were asked to use a five-point Likert scale to express their degree of agreement with the content of each item.

Then, their answers were collected and analysed (Cohen's Kappa correlation test) to identify shared and conflicting viewpoints. The convergence threshold for the answers is set to at least 70%, a value chosen on the basis of previous experiences described in literature [25,26].

12 items (41.4%) reached high convergence (at least 70%) at the first round. The items with a discordant outcome were re-written and represented in the subsequent questionnaire. The process gradually continued aiming at consensus synthesis (14 admission criteria) by the third round. (data not yet published).

Subsequently, the MDDs started the criteria validation as a Gold Standard through the PRUO (Protocollo di Revisione dell' Utilizzo dell' Ospedale) [27] procedure over 1 month (data not yet published).

The PRUO procedure [27], which was introduced for the first time in Italy in 1989 and subsequently adapted and adjusted, is a tool used to assess the appropriateness of the days of hospitalisation. It assumes that, in line with clinical complexity and consequent care needs, there is a hierarchy of healthcare levels based on the intensity of the intervention and on the services rendered. It involves a list of appropriateness criteria for each level of healthcare (which in the project is equivalent to the 14 admission criteria in Intensive rehabilitation facilities defined by the Panel). It also requires an Assessor to check the presence or absence of the criteria in patients’ medical records or other official hospital documents, considering the criterion to be satisfied (presence) when the wording of the criterion listed can be found in the patient’s documentation, in a univocally equivalent form.

The RMSs were blind to the appropriate admission criteria until the end of the research.

### Statistical methods

Construct Validity: upon psychometric validation, the Spearman correlation (ρ) with 95% confidence intervals (CIs) was examined to compare the RCS-E v13 total score and subscales (C-R, N, M, T, E) to BI and CIRS, in order to evaluate case complexity in this context. The strength of the correlation was interpreted according to the guidelines suggested by Evans (1996): .00-.19_very weak, .20-.39_weak, .40-.59_moderate, .60-.79_strong, .80–1.0_very strong [28].

Concurrent Validity: due to the lack of Italian instruments to assess clinical complexity in rehabilitation patients as a gold standard, against which to test concurrent validity, the psychometric validity of the RCS-E v13 was evaluated comparing the 14 Admission Criteria in Intensive rehabilitation facilities (data not yet published) defined with the Delphi Panel Method. The Spearman correlation (ρ) was assessed.

Known-Group Validity: to control the confounding and to examine subgroups and interactions, the ANCOVA analysis has been performed considering as dependent variable the RCS-E v13 total Score, fixed factors, the Gender and Nosological Category and Covariate the Age.

The Kruskall-Wallis test was used to examine the variance between nosological sub-categories.

Dimensionality: the Kaiser-Mayer-Olkin measure and the Bartlett's test of sphericity were assessed to confirm the adequacy for factor analysis, using the AMOS-16 structural equation software programme within SPSS. Exploratory and confirmatory analyses, involving a principal component analysis with orthogonal (Varimax) rotation, were examined in order to evaluate the dimensionality of the scale. The inter-correlation between factors was studied.

The missing data were excluded from the analysis.

The software package “IBM SPSS version 22.0” and “MedCalc version 16.4.3” were used for analyses.

## Results

The data were collected from 1712 Patients, 823 male (48%) and 889 female (52%), mean age 68.34 years old (95% CI 67.69–69.00 years old).

**Fig 1:**
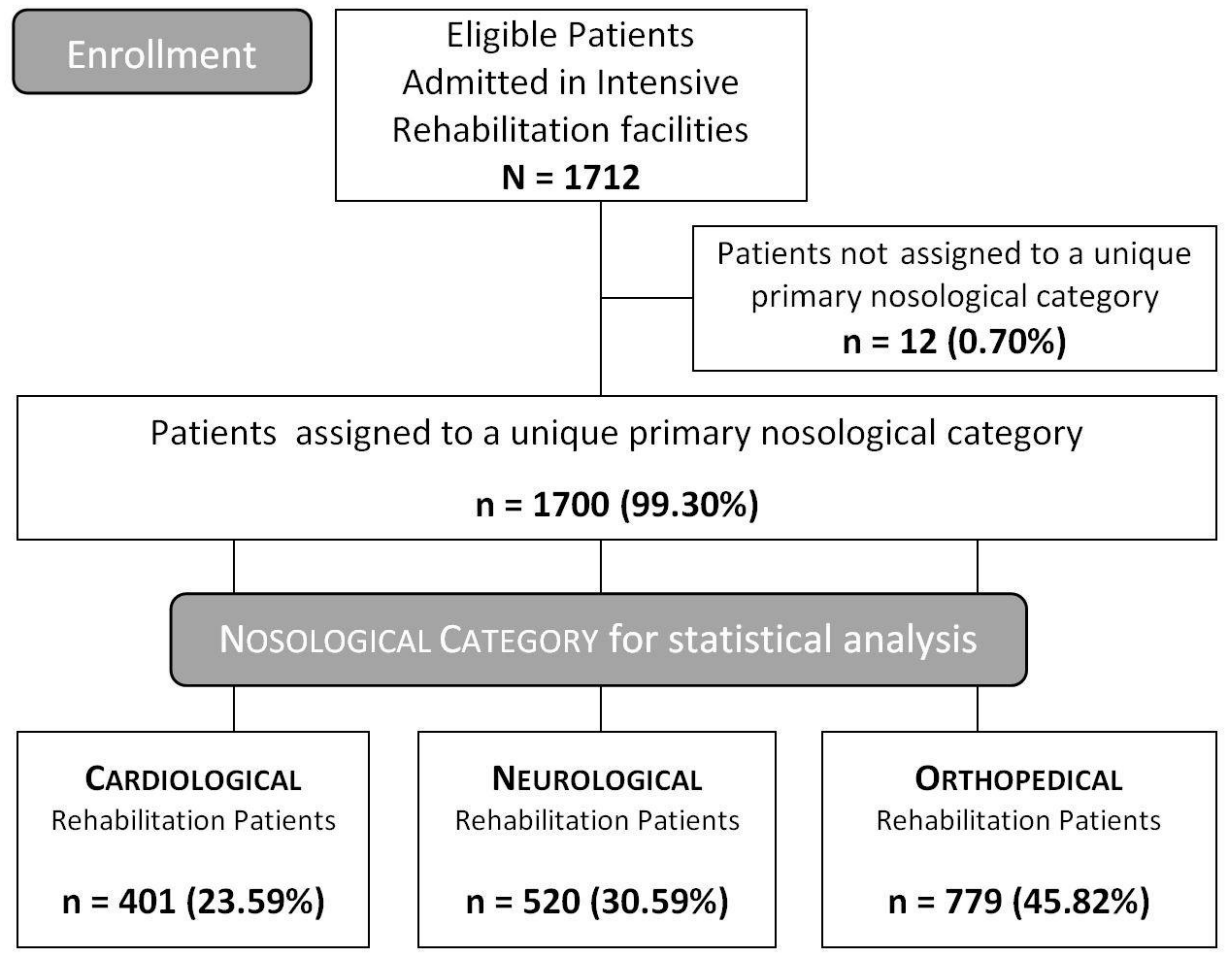
Study flow-chart.

Of these, 1700 (520 NPR, 779 ORP and 401 CRP) were included in the statistical analysis. They were assigned to a unique primary nosological category, without any multiple classifications. See Table 2 for descriptive statistics and the Flow Chart in Fig 1.

**Table 2:** Demographic and clinical characteristic of patients statistically analysed (N = 1700).

		Nosological Category Rehabilitation Patients		
	All	Cardiological	Neurological	Orthopedical
**Characteristic**	n (%), Median [IQR]			
Subject, n	1700	401 (23.6)	520 (30.6)	779 (45.8)
Age, years	70.9 [60.4; 78.2]	71.3 [63.0; 78.7]	69.9 [57.5; 77.6]	71.4 [60.3; 78.9]
Gender, female	882 (51.9)	122 (30.4)	234 (45.0)	526 (67.5)
RCS-E v13				
Care	1.0 [1.0; 2.0]	.0 [.0; 1.0]	2.0 [1.0; 2.0]	1.0 [1.0; 2.0]
Risk	.0 [.0; 1.0]	.0 [.0; 1.0]	1.0 [.0; 1.0]	.0 [.0; .0]
Nursing	2.0 [2.0; 2.0]	2.0 [2.0; 2.0]	2.0 [2.0; 2.0]	2.0 [2.0; 2.0]
Medical	2.0 [2.0; 2.0]	2.0 [2.0; 3.0]	2.0 [2.0; 2.0]	2.0 [2.0; 2.0]
Therapy disciplines	2.0 [1.0; 2.0]	2.0 [2.0; 2.0]	2.0 [2.0; 2.0]	1.0 [1.0; 2.0]
Therapy intensity	2.0 [2.0; 2.0]	2.0 [2.0; 2.0]	2.0 [2.0; 2.0]	2.0 [2.0; 2.0]
Equipment	1.0 [1.0; 1.0]	.0 [.0; 1.0]	1.0 [1.0; 1.0]	1.0 [1.0; 1.0]
*Total*	10.0 [9.0; 11.0]	9.0 [8.0; 10.0]	11.0 [10.0; 12.0]	10.0 [9.0; 10.0]
CIRS				
Severity Index	1.0 [.8; 1.3]	1.2 [.9; 1.5]	1.2 [.9; 1.5]	.9 [.7; 1.1]
Comorbidity Index	5.0 [3.0; 6.0]	5.0 [4.0; 6.0]	5.0 [4.0; 7.0]	4.0 [3.0; 6.0]
*Total*	14.0 [10.0; 18.0]	16.0 [12.0; 20.0]	16.0 [12.0; 21.0]	12.0 [9.0; 15.0]
BI (range 0–100)	40.0 [25.0; 65.0]	77.5 [60.0; 90.0]	25.0 [5.0; 45.0]	30.0 [30.0; 50.0]

Abbreviations: IQR, Inter Quartile Range; RCS-E v13, Rehabilitation Complexity Scale-Extended version 13; CIRS, Cumulative Illness Rating Scale; BI, Barthel Index.

Considering the primary hypothesis, the 5% Type I error rate, the 20% Type II error rate and an expected correlation coefficient r (.07), the studied sample size ensures an 82.3% study power. (G*Power 3.1.9.2).

Table 2 shows the descriptive statistics for RCS-E v13, BI, CIRS of the 1700 patients included in the statistical analysis and assigned to a unique primary nosological category.

About the dimensionality results, all five domains of the RCS-E v13 administered to patients loaded “moderate” to “high” on the first unrotated principal component with loadings ranging from .43 to .72. Only the first two components had eigenvalue > 1, together accounting for 55.57% of the total variance in scores. Parallel Analysis indicated a two-factor solution which was rotated using a Varimax Procedure: the first factor appears to be “Basic Care or Risk—Equipment” which accounts for 31.38% of the variance. These items loaded high (.83-.86) on this factor and low (< .03) on factor 2. The second factor appears to be “Medical—Nursing Needs and Therapy Disciplines”, accounting for 24.19% of the variance. Medical loads .75 on this factor and -.27 on factor 1, Nursing loads .69 and .35, respectively, and, finally, Therapy Disciplines loads .69 on factor 2 and .09 on the other.

The results of the factor analysis suggested that the two-factor model had an excellent fit with the RCS-E v13 scores assessed, as showed with a goodness of fit index (GFI = .999), parsimony Good Fit Index (pGFI = .970) comparative fit index (CFI = .949) and the Bentler-Bonnet Normed Fit Index (NFI = .921). The good fit is confirmed because all indices are above .90 [27] Furthermore, RMSEA = .029 (C.I. 90% .005-.053 p-value .920) and Chi-Square = 9.82 (df = 4, p-value NA) also confirmed the model according to Barrett (2006) [29].

**Fig 2:**
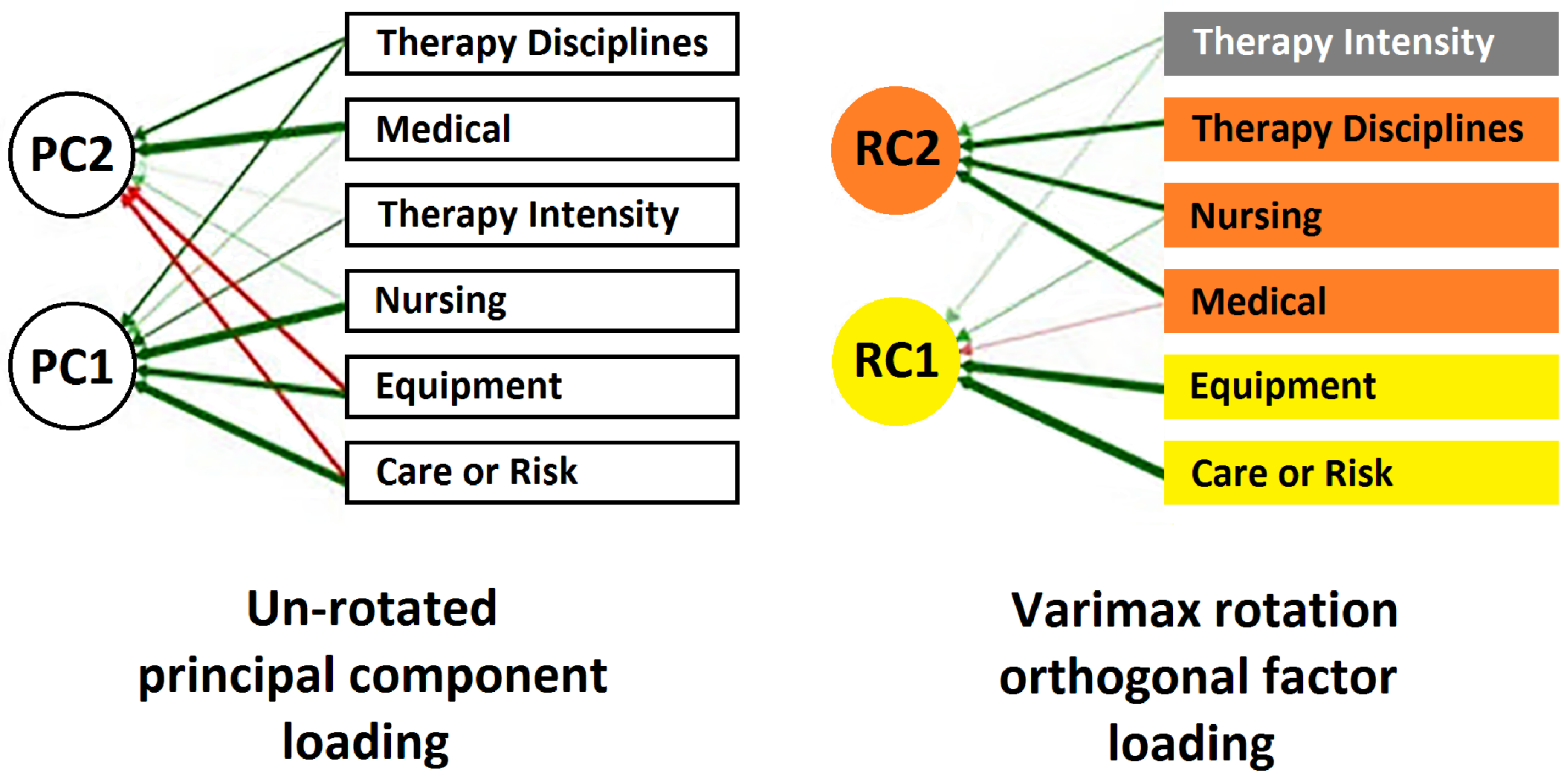
Path diagram of the confirmatory factor analysis of the RCS-E v13.

The covariance between “Basic Care or Risk—Equipment” and “Medical—Nursing Needs and Therapy Disciplines” factors was .12, supporting the different rehabilitation needs involved in each factor. The dimensionality results are reported in Table 3 and the path diagram in Fig 2.

**Table 3:** Results of principal component factor analysis of the RCS-E v13 items.

	Un-rotated principal component loading		Varimax rotation orthogonal factor loading	
RCS-E v13 Items	PC1 Eingenvalue 1.88 % of Variance 31.38	PC2 Eingenvalue 1.45 % of Variance 24.19	RC1	RC2
Care or Risk (C/R)	.686	-.524	.861	.066
Nursing (N)	.721	.276	.354	.685
Medical (M)	.294	.736	-.271	.745
Therapy disciplines (TD)	.466	.507	.009	.688
Therapy intensity (TI)	.430	.116	.243	.373
Equipment (E)	.636	-.536	.832	.024

Abbreviations: RCS-E v13, Rehabilitation Complexity Scale-Extended version 13; PC Principal Component; RC, Rotated Component.

The results about Validity and relationship of the RCS-E v13 with the other measures are reported below. Known-Group Validity: the Kruskall-Wallis test showed the presence of a difference in the RCS-E v13 score among nosological categories (Chi-square 255.89, df 2, p < .001), as reported in Table 4. The ANCOVA showed, although with weak effect, a statistical relationship between the RCS-E v 13 total score, the nosological categories and the age of the sample (η2 .014). The mean value of the RCS-E v13 total score of CRP (mean age 69.7, SD ±11.46) was 9.18; the mean value of NRP (mean age 66.8, SD ±14.65) was 10.97; the mean value of ORP (mean age 68.6, SD ±14.65) was 9.67. These results suggest that being older does not affect the patients’ clinical complexity.

**Table 4:** Comparison of RCS-E v13 scores among the nosological categories of rehabilitation patients.

	Cardiological	Neurological	Orthopedical
Sample, n	401	520	779
Mean	9.18	10.97	9.67
SD	1.34	2.17	1.20
SE	.07	.10	.04
***Kruskal-Wallis test***			
Mean Rank	618.43	1110.07	796.69
Chi-Square = 255.89; df = 2; p < .0001			

Abbreviations: RCS-E v13, Rehabilitation Complexity Scale-Extended version 13; SD, Standard Deviation; SE, Standard Error; df, degree of freedom.

Construct Validity: the RCS-E v13 reported strong negative correlations with the BI (rho = –.62, p < .001). Different correlations were assessed within the subscales, showing strong relation of C (rho = -.75 p < .001), moderate of E (rho = -.54 p < .001), weak of R (rho = -.21 p < .001) and N (rho = -.29 p < .001), and Very Weak of T (rho = -.05 p < .05) and M (rho = .09 p < .001). Furthermore, RCS-E v13 and BI correlations were different for different nosological categories. Correlation was found moderate for CRP (rho = -.58 p < .001) and ORP (rho = -.53 p < .001), and strong for neurological patients (rho = -.62 p < .001).

Weak correlation was found between the RCS-E v13 and the CIRS CTs, CI and SI scores, respectively (rho = .21, p < .001, rho = .21, p < .001, rho = .15, p < .001) and with little or no relation of some subscales like C, M and E, as reported in Table 5. The same results were obtained considering the nosological categories.

**Table 5:** Spearman’s correlation matrix of the RCS-E v13 scores to Admission Criteria, CIRS and Barthel Index.

		RCS-E v13 (Items)						
	RCS-E v13 *Total*	Care	Risk	Nursing	Medical	Therapy disciplines	Therapy intensity	Equipment
Admission Criteria								
(01)	-.117***	-.007	-.347***	.089***	.003	-.338***	-.017	.000
(02)	.*004*	.001	.018	.013	-.009	.021	-.001	-.018
(03)	.*011*	.047	-.039	.049*	.078**	-.028	-.091***	.006
(04)	-.*010*	.023	-.039	-.020	.012	-.032	.047	.000
(05)	.242***	.208***	.156***	.076**	-.036	.156***	.005	.194***
(06)	.332***	.320***	.288***	.072**	-.181***	.175***	.036	.359***
(07)	.272***	.230***	-.039	.154***	.187***	.076**	.099***	.119***
(08)	.146***	.113***	.107***	.115***	.048*	.137***	.089***	.110***
(09)	-.070**	-.038	-.053*	-.022	.042	-.056*	-.011	-.065**
(10)	.150***	-.026	.323***	.018	-.014	.387***	.043	.021
(11)	.050*	.030	.085***	.039	.007	.041	-.001	.021
(12)								
(13)	.054*	.049*	.073**	.037	.019	.027	-.056*	.037
(14)								
CIRS								
Severity Index	.154***	.014	.118**	.158***	.079**	.295***	.003	-.069**
Comorbidity Index	.211***	.133***	.107***	.218***	.052*	.199***	.021	.013
*Total*	.208***	.073**	.171***	.197***	.046	.317***	.027	-.033
BI (range 0–100)	-.616***	-.747***	-.207***	-.288***	.094***	-.050*	-.069**	-.538***

* p < .05,

** p < .01,

*** p < .001

Abbreviations: Admission Criteria, (12) and (14): always absent in the medical records; RCS-E v13, Rehabilitation Complexity Scale-Extended version 13; CIRS, Cumulative Illness Rating Scale; BI, Barthel Index.

Concurrent Validity: the RCS-E v13 total Score demonstrated an overall correlation with most criteria, even if the rho resulted poor-modest (.05 to .27), thus confirming the concurrent validity of the tool. No statistically significant correlation was found between the RCS-E v13 score and criteria 2 (favourable rehabilitative prognosis), 3 (nursing access at least 3 times a day), 4 (daily need for medical intervention), 12 (hospitalisation due to the inability to move from the house to the gym) or 14 (treatments integration); these criteria were found always or almost always absent (2, 12, 14) or present (3 and 4), in the medical records. However, within the subscales, there were different correlations as reported in Table 5.

## Discussion

The objective of this work is demonstrating the psychometric validity of the RCS-E v13 scale proposed to a sample of 1712 patients hospitalized in 8 different intensive rehabilitation centres of the Emilia Romagna Region. The statistical reliability of the pre-final version of the tool, cross-culturally adapted and investigated in accordance with the relevant international guidelines [30,31], was shown on a sample of 51 different patients previously investigated [22].

Currently, the absence, in Italy, of a shared tool for the evaluation of clinical complexity and the use of different evaluation tools among facilities have led to the proposal, to be defined through a scientifically-validated method, of the appropriate criteria for hospitalisation in intensive rehabilitation units that can be adopted as reference Gold standard (data not yet published). The Delphi Panel [24–26,32] method was chosen. Through a Delphi Methodology, a national panel of experts in rehabilitation was called to examine the admission criteria of the 8 facilities participating in the project. The panel drew conclusions about the collected information and compared it to the literature in order to prepare a First Italian list of criteria (as standards) for appropriate admission to intensive rehabilitation facilities (data not yet published). The Panel produced a list of 14 general criteria (data not yet published) that can be adopted for all three nosological categories (NRP, CRP, ORP) studied (S1 Appendix). Criteria exploring all elements of clinical practice, such as the medical, nursing and time (3 months) areas, social aspects, rehabilitation/assistance planning and multidisciplinary path (n 5-6-7-8), would support the definition of tailor-made Rehabilitative Projects.

The list was then validated through the PRUO (Protocollo di Revisione dell'Utilizzo dell'Ospedale) [27] procedure. To minimise/prevent biases, ensure scientific validity of the collected data and their reliability to be used as a gold standard for admission to rehabilitation, randomization and blinding approaches were be used. The centres will be masked from the list of criteria (data not yet published) that will be known by the MDDs only.

Even though low, the correlation of these criteria with the RCS-E v13 seems suitable for investigation because, it can represent the relation between the patient’s complexity and the relevant rehabilitative objectives. The correlation of the subscales composing the RCS-E v13 and the differences between nosological categories seem to support this interpretation. Overall, these results have shown the concurrent validity of the tool. The Results of Known-Group Validity have shown that there are no statistical differences between nosological categories when the data are adjusted by age. The oldest patients are the CRP group who obtained the minor RCS-E v13 score. This shows that being older does not entail being clinically more complex. This unexpected result requires further investigations. The construct validity of the RCS-E v13 has been verified by correlating it with the other instruments, as described below. The comparison of the RSC.E v13 to BI suggests that the disability domain is a strong indicator of care needs, a moderate one for Equipment and a very week indicator of Medical and Therapeutic Needs, as resulted from the Original English Scale [17] and the Italian RCS-E v12 [21], retrospectively validated in the work by Galletti et al. [21]. Weak correlation was found with the newly added subscale Risk. The same general results have been confirmed for the neurological patients, while, for the cardiologic rehabilitative category, the disability degree, on average higher than the others, is a strong indicator of Care Needs only, a moderate one for equipment and a weak indicator for Risk. It is not an indicator of T and R in the orthopaedic patients. Weak correlation was found among RCS-E v13, CTs and the two indices SI and CI of the scale, which were even less correlated in the CRPs, as reported also in Galletti et al. [21], confirming the idea that probably CIRS is not a tool that directly measures the patient’s rehabilitative needs. These results appear to support the hypothesis that the comorbidity, expressed by the CIRS scale, summarised in the two indicators CI and SI, is not, therefore, a domain necessarily related to the clinical complexity of the rehabilitative patient. The factor analysis suggested that the Italian version of the scale has two different components (“Basic Care or Risk—Equipment” and “Medical—Nursing Needs and Therapy Disciplines”). This implies that each one of the scale 5 domains has a different impact in terms of rehabilitation requirements and the 5 domains together provide accurate profiling of rehabilitation needs.

### Limits and strengths

In this research, some limits may be recognized:

A limit of the research is the inability to compare the scale to tools currently shared for direct evaluation of clinical complexity because, for the time being, they do not exist in the Italian rehabilitative scenario. However, this situation has generated the first national attempt to identify the shared intensive rehabilitation hospitalisation criteria.

It is also true, however, that the Gold standard defined by the National Panel was the result of the work of both clinical and administrative staff engaged in rehabilitation. The comparison to a totally clinical tool, such as the RCS-E v13 may have influenced the correlation indices that were not always found strong. However, the fact that the RCS-E v13 correlates with the criteria as well as with the BI and CIRS scales seems to support the validity of the tool as a clinical complexity indicator.

Another limit of this study is the case-mix investigated, which, in part, differed from the validation study of the original scale and from the pre-final 13 Italian version, in that it included also cardiological and orthopaedic patients. Nevertheless, the use of a homogeneous group of pathologies and the size of each one allowed the tool to validated for the measurement of rehabilitation complexity in patients having different rehabilitation needs, as previously demonstrated in Hoffmann et al. [33] and Galletti et al [21].

The study was conducted on only one intensity level of the rehabilitative hospitalisation, the intensive one. However, it is believed that the choice of a multicentre investigation, together with the involvement of patients belonging to different nosological categories for which disability and comorbidity are not necessarily equal, may be a strong point.

Another strong point is the perspective design of the study which could confirm that the ease and the speed in administering the RCS-E v13 make it a tool that can be effectively introduced in clinical settings.

## Conclusions

Taking into consideration objectives, limits, range of analyses, similar results of other recent studies and other relevant evidence, we may conclude that version 13 of the Italian RCS-E has proved a tool that can be used for the evaluation of rehabilitative patients’ clinical complexity.

In order to evaluate the scale responsiveness and confirm its routine application in the national territory, further studies would be useful adopting administration not only at hospitalisation but also at discharge and in more levels of care intensity other than the intensive ones.

Efficient rehabilitation intervention requires rehabilitation settings that meet patient needs.

This study may provide a model for optimal use of rehab beds, for redefining reimbursement rates and assessing the required rehabilitation resources considering all three dimensions (disability, comorbidity and clinical complexity) as required by the Guidelines [1]. The 8 rehabilitative centres involved are representative of the Italian range of rehab facilities and patients; all this is expected to produce results fit to be used throughout the NHS to assess rehab needs.

Validated tools supporting referral to rehabilitation would make the admission process more appropriate and sharable. Furthermore, not only would they contribute in reducing national variability, but they would also improve clinical appropriateness.

## Supporting information

S1 Appendix List of Admission Criteria in intensive rehabilitation facilities. (DOCX)

S1 Table Database of the study. (XLSX)
