# Supplementary material for: Psychometric validation of the Italian Rehabilitation Complexity Scale-Extended version 13
Source: PLoS One. 2017 Oct 18;12(10):e0178453. doi: 10.1371/journal.pone.0178453 (PMC5646770; doi:10.1371/journal.pone.0178453)
Supplement: S1 Appendix — (DOCX) [file pone.0178453.s001.docx]

| **Criteri di Appropriatezza di Ricovero in Riabilitazione Intensiva** | |
| --- | --- |
| **01** | the patient is in a phase of clinical stability (cardiocirculatory, respiratory, metabolic, infectious, etc. stability) |
| **02** | the patient’s rehabilitation prognosis is favourable |
| **03** | the patient requires daily nursing assistance (at least 3 times every 24 hours) |
| **04** | the patient requires daily medical care. |
| **05** | the patient requires assistance/rehabilitation treatment concerning upper body control in posture transfers (bed - chair; chair - standing) |
| **06** | the patient requires assistance/ rehabilitation treatment concerning the management of nutrition and/or urination/ defaecation |
| **07** | the patient requires an integrated multidimensional rehabilitation strategy (simultaneous presence of at least 2 of the following rehabilitation strategies: motor, cognitive, swallowing, behavioural, occupational, sight) |
| **08** | 8. the patient uses invasive medical devices (cannula, PEG, etc.) for which a weaning project is needed. |
| **09** | persistence of certain criteria (indicated in points 3 to 8) for no more than 3 months |
| **10** | intensive rehabilitation follows admission for an acute event. |
| **11** | the patient cannot be managed at home, regardless of symptom severity |
| **12** | the patient is unable to travel from his/her home to the gym |
| **13** | the patient is unable to manage his/her medication personally |
| **14** | the rehabilitation project cannot be implemented without a significant need for integration between the various treatment administered |

**S1 Appendix. List of Admission Criteria in intensive rehabilitation facilities**
